# HALO CleanSpace PAPR evaluation: Communication, respiratory protection, and usability

**DOI:** 10.1017/ice.2022.71

**Published:** 2023-02

**Authors:** Irene Ng, Keat Lee, Benjamin Kave, Michael Kluger, Camille Paynter, Reny Segal, Roni Benjamin Krieser, Paul Mario Mezzavia, Shan Hung, Yinwei Chen, Teresa Sindoni, Daryl Lindsay Williams

**Affiliations:** 1 Department of Anaesthesia and Pain Management, Royal Melbourne Hospital, Parkville, Australia; 2 University of Melbourne, Parkville, Victoria, Australia; 3 Department of Speech Pathology, The Royal Melbourne Hospital, Parkville, Australia; 4 Department of Audiology and Speech Pathology, University of Melbourne, Parkville, Australia; 5 Department of Critical Care, Faculty of Medicine, Dentistry and Health Sciences, University of Melbourne, Parkville, Australia; 6 Anaesthetic & Recovery, Royal Melbourne Hospital, Parkville, Australia

## Abstract

**Objective:**

To evaluate a relatively new half–face-piece powered air-purifying respirator (PAPR) device called the HALO (CleanSpace). We assessed its communication performance, its degree of respiratory protection, and its usability and comfort level.

**Design and setting:**

This simulation study was conducted at the simulation center of the Royal Melbourne Hospital.

**Participants:**

In total, 8 voluntary healthcare workers participated in the study: 4 women and 4 men comprising 3 nursing staff and 5 medical staff.

**Methods:**

We performed the modified rhyme test, outlined by the National Institute for Occupational Safety and Health (NIOSH), for the communication assessment. We conducted quantitative fit test and simulated workplace protection factor studies to assess the degree of respiratory protection for participants at rest, during, and immediately after performing chest compression. We also invited the participants to complete a usability and comfort survey.

**Results:**

The HALO PAPR met the NIOSH minimum standard for speech intelligibility, which was significantly improved with the addition of wireless communication headsets. The HALO provided consistent and adequate level of respiratory protection at rest, during and after chest compression regardless of the device power mode. It was rated favorably for its usability and comfort. However, participants criticized doffing difficulty and perceived communication interference.

**Conclusions:**

The HALO device can be considered as an alternative to a filtering face-piece respirator. Thorough doffing training and mitigation planning to improve the device communication performance are recommended. Further research is required to examine its clinical outcomes and barriers that may potentially affect patient or healthcare worker safety.

Healthcare workers caring for patients with COVID-19 are required to wear appropriate respiratory protective devices as part of a defined respiratory protection program.^1–6^ N95/P2 filtering face-piece respirators (FFRs) are the most widely used respirators in healthcare settings.^1^ However, given the concerns about unequal worldwide distribution, limited stockpile, and the need for prolonged use of FFRs, there is renewed interest in reusable powered air-purifying respirators (PAPRs).^7–10^

Tight-fitting full-face and half-face PAPRs protect the user through a combination of creating a face seal, filtering out air contaminants through high-efficiency particulate air (HEPA) filters, and providing purified airflow under positive pressure via a battery-operated blower unit.^10,11^ Tight-fitting PAPRs provide higher assigned protection factors (APFs)^12,13^ and are often rated more comfortable for prolonged wear^14^ than N95/P2 FFRs.

Although full–face-piece PAPRs achieve a superior face seal with a higher designated APF^15^ and have the added benefit of splash and eye protection, half–face-piece respirators are generally preferred in healthcare possibly because of ease of use, comfort, and lower cost. A relatively new lightweight and compact half–face-piece PAPR called the HALO (CleanSpace Technology, St Leonards, NSW, Australia) has recently been developed and registered for clinical use.^16,17^ Studies on the efficacy and usability of this device are limited. One recent study completed a fit test on 20 participants, most of whom found the device easy to don and doff and comfortable to wear.^18^ The lack of clinical or simulated workplace studies on half–face-piece PAPRs has resulted in considerable international variation in the APFs for this class of respirator.^3,4,12,13^

We evaluated the half–face-piece HALO PAPR device (1) by exploring communication performance using the modified rhyme test (MRT)^19^; (2) by investigating respiratory protection level using quantitative fit test (QNFT) and simulated workplace protection factor (SWPF) studies; and (3) by assessing usability and comfort via a survey.

## Methods

This simulation study was conducted at the simulation center of the Royal Melbourne Hospital. The project was approved by the Melbourne Health Human Research Ethics Committee (QA no. 2020197). Both the MRT and QNFT assessments were conducted in an isolation room, with neutral air pressure, 20 air exchanges per hour, and steady temperature and humidity.

In total, 8 voluntary healthcare workers from the Royal Melbourne Hospital were invited to participate in this study. Participants were all fluent in English, with no obvious or strong accents and were certified for the delivery of basic life support. We excluded individuals with facial hair, concurrent respiratory disease or symptoms, claustrophobia or anxiety when wearing the HALO device, hearing impairment, back pain, or wrist pain.

Before the study began, all participants received an online education package regarding the safe use of the HALO device, including donning, seal check, and doffing techniques. Participants were fit checked to the appropriate size of the HALO PAPR mask. On the day of the simulation, all participants donned and fit checked the HALO device according to the manufacturer’s instructions.^17^ A trained superuser, who was a designated HALO device educator in our institution, confirmed correct fit before each of the assessments. This superuser also ensured that the harness holding the device was sitting securely on the participant’s head and that the neck brace fit firmly on the power unit to provide extra neck support.

### Part 1: Communication performance assessment

**Fig. 1.**
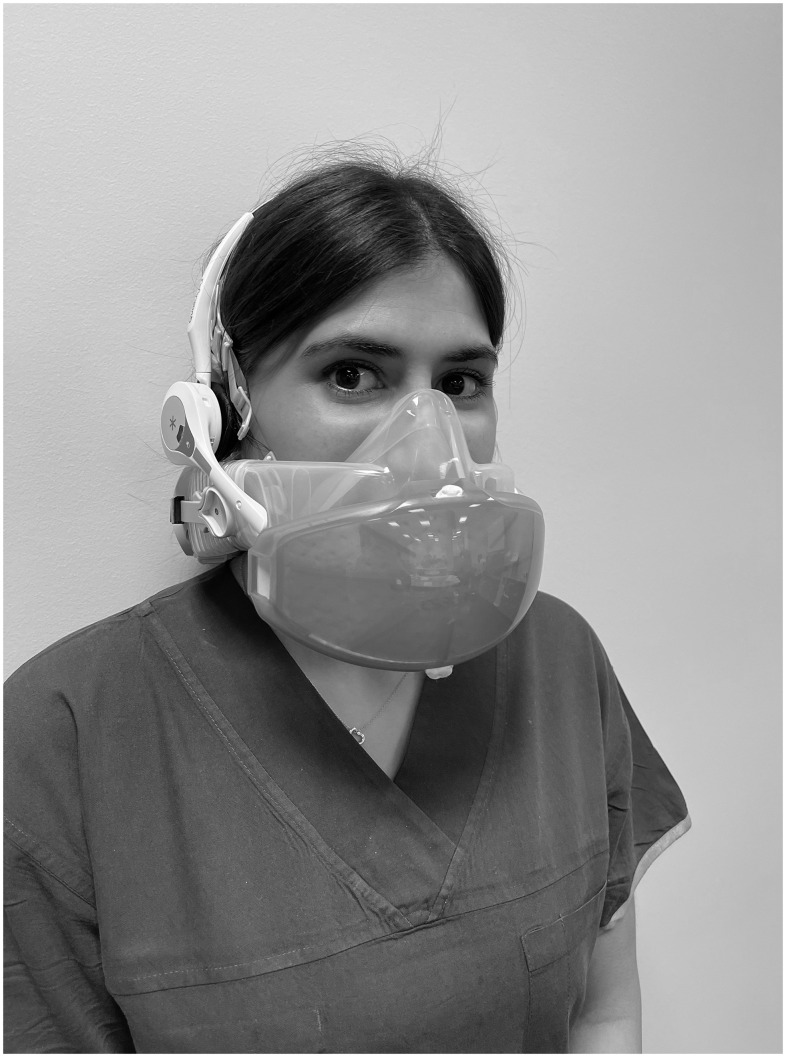
A healthcare worker wearing a HALO CleanSpace powered air-purifying respirator and a wireless communication headset device.

We assessed the communication performance of the HALO device using the MRT, as outlined by the National Institute for Occupational Safety and Health (NIOSH) in the standard testing procedure.^19^ The 8 volunteers were divided into 2 groups: a listener group with 3 participants, and a speaker group with 5 participants (at least 1 woman and 1 man in each group). We evaluated the listeners’ ability to comprehend 50 random single-syllable words (Appendix 1) from each of the 5 speakers. We added 20 random, commonly used medical phrases (Appendix 2) that were not in the NIOSH protocol. We tested 3 experimental conditions: (1) speakers not wearing the HALO device; (2) speakers wearing the HALO device with power on; and (3) speakers wearing the HALO device with power on, plus both speakers and listeners wearing a wireless communication headset (Pro 11 Quail Digital, Dallas, TX) (Fig. 1). Thus, we conducted 15 communication performance assessment trials.

**Fig. 2.**
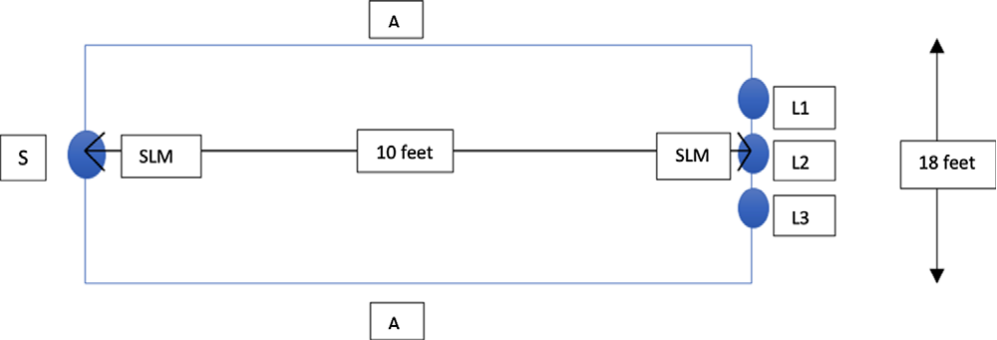
Diagram to show the room setup for the modified rhyme test. Note. S, speaker; L1, listener 1; L2, listener 2; L3, listener 3; SLM, sound level meter; A, amplifier.

The room setup was shown in Figure 2. A background noise in the frequency range of 20–50 kHz was transmitted through 2 amplifiers. Two precalibrated sound-level meters (Optimus, Cirrus Research, North Yorkshire, UK) were used to ensure that the background noise was at 60±2 dBA and that the speaker’s voice level was at 75–85 dBA. Immediate feedback was provided by a test administrator if required.

Each speaker took randomized turns reading 50 words out loud, using the phrase, “The word is (list word)” from the given word list of 15 random word lists (with no repeats), followed by 20 medical phrases randomly selected from a pool of 180 phrases. The 3 listeners, sitting next to each other, selected the word they perceived to be spoken from 6 possible words on the provided answer sheet (Appendix 3) and wrote down the medical phrases as heard. A second test administrator recorded any words spoken incorrectly.

All conditions of the 15 trials were randomly assigned, including the individual word and medical phrase list, the order of speakers, whether the speaker was wearing the HALO device, and whether the speaker was wearing a communication headset. The intelligibility of single-syllable word reading was measured by calculating the adjusted score, performance rating, and overall performance rating according to NIOSH protocol.^19^ According to NIOSH criteria, the overall performance rating of a PAPR is required to be at least 70%. We also compared the percentage of adjusted correct words perceived among the 3 experimental conditions. We measured the intelligibility of medical phrase reading by calculating the percentage of correct words and correct meanings perceived. Correct meaning was defined as having the key words recorded correctly or using a synonym instead. Again, we compared these results among the 3 experimental conditions.

### Part 2: Respiratory protection assessment

We assessed the respiratory protection of the HALO device by performing QNFT and SWPF studies on each of the 8 participants. According to the Australian and New Zealand fit-testing standard,^4^ all participants were required to achieve a fit factor of at least 100 at baseline with the HALO power turned off (ie, negative pressure mode) to pass the test.

The QNFT was performed by fit-test operators, who were qualified by a certified training program, using the ambient aerosol-condensation nuclei-count method on a Portacount machine (PortaCount Pro+ 8048, TSI, St Paul, MN). We conducted the QNFTs according to the US Occupational Safety and Health Administration (OSHA) fast half-mask respirator protocols^20^ with 4 conventional exercises: (1) bending over at the hips and returning to upright repeatedly while taking 2 breaths in each position for 50 seconds; (2) jogging on the spot for 30 seconds; (3) moving the head from side to side for 30 seconds; and (4) flexing and extending the neck for 30 seconds.

After pass the QNFT, each participant with the HALO device powered it off, then proceeded to the SWPF study by performing continuous chest compressions for 2 minutes on a Resusci Anne mannequin (Laerdal Medical, Stavanger, Norway). One investigator, a qualified advanced life support instructor, was present to provide direct feedback to the participants on the quality (speed and depth) of the chest compressions. We measured the SWPF using the Portacount machine during 2 minutes of chest compressions. The participants then immediately undertook another QNFT in power off (ie, negative pressure) mode. We then repeated the entire testing process, with the HALO power turned on, including baseline, during, and immediately after chest compression.

The fit factor and the SWPF were calculated using the Portacount machine, by dividing the concentration of the particles in ambient air outside the mask by that inside the mask. We examined the changes of fit factors or SWPFs throughout the 3 phases: before, during, and immediately after chest compressions, for each condition, with the HALO power on or off. We also compared the fit factors or SWPFs between power on and power off with the HALO device.

### Part 3: Usability and comfort assessment

All participants were asked to complete a usability and comfort survey of the HALO device (Appendix 4). Most questions were in either 3-point or 5-point Likert-scale format.

### Statistical analysis

Sample size of 8 was chosen according to the NIOSH protocol, to allow calculation of the MRT results.^19^ Descriptive statistics were used to present the MRT outcomes, the QNFT or SWPF scores, and the usability and comfort assessment results. The Friedman test was used to compare continuous data (eg, percentage of correct words or correct meanings, and fit factors or SWPF) among groups >2 (ie, the unmasked group, the HALO group and the HALO with headset group in the communication performance assessment; and before, during and after chest compression in the QNFTs). This procedure was followed by a pairwise comparison using Wilcoxon signed-ranks test between each of the 3 possible pairs. *P* < .05 was considered statistically significant. Statistical analysis was performed using Stata version 13.0 software (Statacorp, College Station, TX).

## Results

We recruited 8 healthcare workers (4 women and 4 men) for this study; 3 were nursing staff and 5 were medical staff. All achieved a fit factor of at least 100 for their baseline QNFT with the HALO device power off. Their overall average height was 167.6±10.9 cm and their overall average weight was 66.1±13.4 kg. Also, 3 participants used a small-sized mask and 5 required a medium size. None of the participants had any physical limitation that would interfere with the study.

### Part 1: Communication performance assessment

**Fig. 3.**
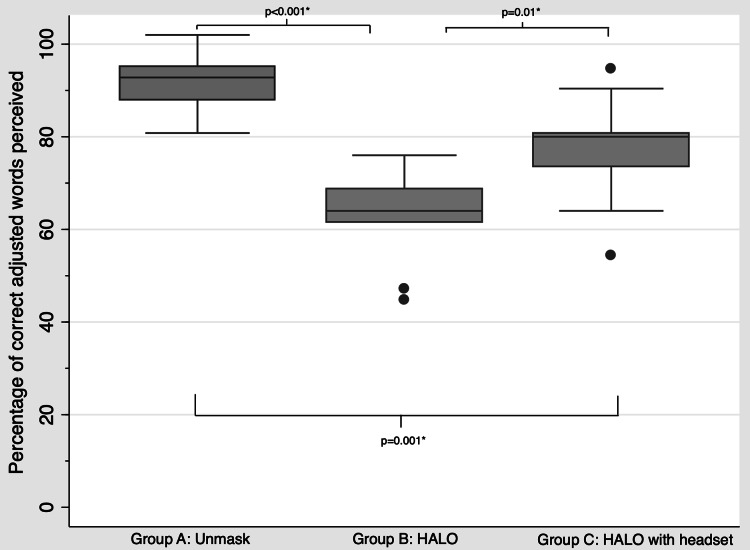
Comparison of percentage of adjusted correct words perceived in the modified rhyme test among the 3 groups where the speakers were (A) unmasked, (B) wearing the HALO device, or (C) wearing the HALO device with a wireless communication headset.

The overall communication performance rating of the HALO device was 69.5% in the MRT. This rating improved to 84.3% when both the speaker and the listener wore the wireless communication headset. We detected a significant reduction in the percentage of adjusted correct words perceived when the speakers were wearing the HALO device compared to when they were unmasked (Fig. 3). However, comprehension significantly improved with the addition of the wireless communication headset.

Regarding the intelligibility of random medical phrases, 100% (IQR, 100%–100%) of the words and 100% (IQR, 100%–100%) of the meanings were correctly perceived when the speakers were unmasked. These rates significantly decreased to 98% (IQR, 94%–99%; *P* = .002) and 95% (IQR, 85%–100%; *P* = .004), respectively, when the speakers were wearing the HALO device. There were no significant changes after the addition of the headset, with 97% (IQR, 95%–100%; *P* = .549) of correct words and 95% (IQR, 85%–100%; *P* = .436) of correct meanings perceived.

### Part 2: Respiratory protection assessment

The median fit factors and SWPFs were >1,000 throughout the entire study (Table 1 and 2). There were no significant changes in the overall fit factors or SWPFs obtained by the participants before, during and immediately after chest compressions, regardless of whether the participant had the HALO device power turned on or off (Table 1). Also, there were no significant differences in the overall and individual scores between HALO power turned on and off (Table 2).

**Table 1.** Comparison of the Overall Quantitative Fit Factors or Simulation Workplace Protection Factors (SWPFs) Obtained by Participants; and the Proportion of Participants Achieving an Overall Fit Factor or SWPF >100^a^

Power	Fit Factor or SWPF	Before Chest Compression	During Chest Compression	After Chest Compression	*P* Value
Off	Overall score, median (IQR) [range]	1,869 (617–4333) [265–7,407]	1,748 (378–6,881) [84–12,170]	1,243 (669–3,881) [130–9,187]	.69
	Score >100, %	100	87.5	100	.37
On	Overall score, median (IQR) [range]	3,576 (2,128–6,109) [428–8,522]	4290 (2,048–4,931) [502–6,381]	4,135 (2,913–6,890) [496–7,720]	.09
	Score >100, %	100	100	100	…

a Before, during and after chest compression, while wearing the HALO device with the power on and power off.

**Table 2.** Comparison of Overall and Individual Quantitative Fit Factor or Simulation Workplace Protection Factor (SWPF) Between Power on and Power Off With the HALO Device, Before and After Chest Compressions

Chest Compression	Fit factor or SWPF	HALO Power Off, Median (IQR) [Range]	HALO Power On, Median (IQR) [Range]	*P* Value
Before	Overall score	1,869 (617–4,333) [265–7,407]	3,576 (2,128–6,109) [428–8,522]	.40
	Individual score			
	Exercise 1	4,773 (1,653–7,813) [434–14,131]	4,127 (2,057–6,105) [430–7,334]	.78
	Exercise 2	1,038 (306–2,469) [207–4,262]	2,961 (1,724–4,845) [413–7,395]	.12
	Exercise 3	3,650 (952–4,557) [241–6,293]	3,732 (1,861–6,490) [455–11,931]	.16
	Exercise 4	4,995 (846–11,940) [263–14,695]	5,600 (2,675–9,108) [417–10,104]	.78
During	Overall score	1,748 (378–6,881) [84–12,170]	4,290 (2,048–4,931) [502–6,381]	.83
After	Overall score	1,243 (669–3,881) [130–9,187]	4,135 (2,913–6,890) [496–7,720]	.16
	Individual score			
	Exercise 1	4,374 (1,165–8,215) [247–9,362]	5,041 (3,085–6,643) [527–7,475]	.89
	Exercise 2	1,809 (491–3,305) [98–6,241]	4,025 (2,583–5,820) [470–8,313]	.09
	Exercise 3	2,238 (1,045–5,087) [108–10,133]	4,215 (2,322–5,906) [487–7,145]	.26
	Exercise 4	4,020 (503–12,140) [138–14,356]	5,097 (2,885–9,397) [504–11,398]	.89

### Part 3: Usability and comfort assessment

The usability and comfort results are shown in Table 3. Of the 8 participants, 7 rated the overall assessment as good or very good. Most found the HALO device easy to don, with good breathability and firmness. The main issues were difficulty to doff and communicate; 2 participants specifically complained about noise from the device.

**Table 3.** Usability and Comfort Assessment of the HALO Device

Variable	Difficult	Somewhat difficult	Easy
Ease of donning	0/8	1/8	7/8
Ease of doffing	1/8	4/8	3/8
	Poor	Average	Good
Breathability	0/8	2/8	6/8
Communication performance	2/8	6/8	0/8
	Too loose	Too tight	About right
Firmness of fit	0/8	2/8	6/8
	Likert scale 1–2^a^	Likert scale 3^a^	Likert scale 4–5^a^
Seal rating	0/8	0/8	8/8
Overall comfort	0/8	5/8	3/8
Overall assessment	0/8	1/8	7/8

a Five-point Likert scale: 1, very poor; 2, poor; 3, average; 4, good; 5, very good.

## Discussion

The provision of high-quality RPE during the COVID-19 pandemic has been a significant challenge for healthcare systems around the world.^21^ To address the issues of supply constraints and sustainability, it is essential to consider reusable and effective alternatives to FFRs, such as PAPRs.^22^ The HALO device is a relatively new half–face-piece PAPR, which has been marketed specifically for healthcare as the most lightweight PAPR available. It potentially offers healthcare workers better maneuverability and comfort than other conventional PAPRs.^23,24^ This study is the first to conduct a multifaceted review of the device. The HALO device met the NIOSH requirement for speech intelligibility. It provided adequate respiratory protection at rest, during, and after chest compression regardless of the power mode. Most of our participants found the device easy to use and comfortable to wear.

Previous research has demonstrated that respirators decrease speech intelligibility by anywhere between 1% and 17%, depending on the type of respirator.^25,26^ Although the HALO device had a significant negative impact on speech intelligibility (as shown in this study), it met the requirement of achieving an overall performance rating of 70%. In Radonovich’s study,^25^ a simple surgical mask at a distance of 2.1 m (7 feet) led to an MRT accuracy rate of 86%. When compared to our study, which was performed at a distance of 3 m (10 feet), the performance of the HALO with a communication headset was nearly equivalent to that of a simple surgical mask.

The HALO device performed well with respect to the intelligibility of random medical phrases, with ≥95% accuracy, regardless of whether the communication headset was used or not. This high level of intelligibility could potentially be due to the influence from the contextual information associated with sentences, which may have assisted participants to predict the words being read.^27,28^ This study reminded us not only to be cognizant of the potential risk of compromising patient care due to decreased speech intelligibility but also to consider strategies to mitigate this negative impact, such as use of technology, modification of verbal dialogue, and use of nonverbal communication.

Importantly, our results demonstrated that the HALO device provided high-quality respiratory protection, with the median fit factor or SWPF > 1,000 throughout, in both power-on and power-off modes. This high level of protection persisted during the conduct of chest compression, which carried a risk of seal break from repetitive forceful movements.

In the power-on mode, which is the default use, the median SWPF during chest compression was 4,290. In the power-off mode, which would only be used as a back-up in the case of power failure, the median SWPF decreased to 1,748. Several previous studies demonstrated insufficient respiratory protection from FFRs during chest compression,^29,30^ whereas for PAPRs, findings were conflicting.^31,32^ However, our study has demonstrated respiratory protection well above the various designated APFs from around the world (ie, 50 from OSHA, 40 in Europe and 10 in Australia).^4,12,13^

Encouragingly, the overall assessment and comfort level of the HALO device were rated positively by our participants. The 2 apparent issues commonly reported were difficulty doffing and noise from the device affecting communication. Difficulty of doffing is a known drawback of PAPR use when compared to FFRs,^32^ and this finding emphasizes that effective donning and doffing training is important when implementing a PAPR program in a healthcare institution.^33^ Similarly, the perceived communication interference was consistent with our speech intelligibility findings, and mitigation methods need to be considered when implementing a PAPR program.

This study had several limitations. Primary among these was the low number of participants. We complied with the sample size requirement according to the NIOSH protocol. Although we demonstrated strong performance of the HALO device in respiratory protection, it might be beneficial to repeat the SWP studies in a broader population to capture a larger data set. We recommend a larger usability study to examine any factors affecting HALO utilization in the broader healthcare workforce. Another limitation was the lack of blinding for both listeners in the intelligibility test and fit testers in the QNFT, but this was not pragmatic for a study of this nature. Finally, we did not conduct formal hearing assessments on our participants, which should be considered in future studies to further improve the quality of the findings.

Our study demonstrated that the HALO half–face-piece PAPR is a relatively safe device, by meeting NIOSH minimum standard for speech intelligibility, and by providing consistent and adequate level of respiratory protection at rest, during and after chest compression regardless of the device power mode. Overall, the HALO PAPR was rated favorably in terms of its usability and comfort. However, thorough doffing training and mitigation planning to improve communication performance should be considered if a HALO PAPR program is to be implemented in a healthcare setting. We recommend further research to examine clinical outcomes associated with the use of the device and to investigate any barriers that may potentially affect patient or healthcare worker safety.

## Supplementary material

For supplementary material accompanying this paper visit https://doi.org/10.1017/ice.2022.71. click here to view supplementary material
