# Supplementary material for: HALO CleanSpace PAPR evaluation: Communication, respiratory protection, and usability
Source: Infect Control Hosp Epidemiol. 2022 Apr 1;44(2):295–301. doi: 10.1017/ice.2022.71 (PMC9929704; doi:10.1017/ice.2022.71)
Supplement: Supplementary file 1 [file S0899823X2200071Xsup.zip › S0899823X2200071Xsup001.pdf]

Appendix 3: Listener answer sheet sample

WORD LIST 7-12															
**1**	sick	pick	wick	**14**	sag	sad	sap	**27**	sud	sun	sup	**40**	came	cave	case
	tick	kick	lick		sat	sass	sack		sung	sum	sub		cake	cape	cane
**2**	beat	meat	heat	**15**	sin	sip	sick	**28**	led	bed	wed	**41**	same	tame	game
	neat	feat	seat		sit	sill	sing		shed	fed	red		name	fame	came
**3**	puff	pup	puck	**16**	gold	fold	hold	**29**	lot	tot	pot	**42**	toil	foil	oil
	pus	pub	pun		sold	cold	told		got	not	hot		soil	boil	coil
**4**	shook	book	took	**17**	but	bug	bus	**30**	dub	duck	dug	**43**	fin	fill	fit
	hook	look	cook		buff	bun	buck		dung	dud	dun		fib	fig	fizz
**5**	tip	lip	dip	**18**	lame	late	lake	**31**	pip	pin	pick	**44**	cuff	cub	cud
	rip	sip	hip		lay	lane	lace		pig	pill	pit		cuss	cut	cup
**6**	ray	raze	rate	**19**	gun	sun	fun	**32**	seep	seek	seed	**45**	reel	peel	keel
	rake	race	rave		nun	run	bun		seethe	seem	seen		eel	feel	heal
**7**	rang	hang	gang	**20**	rust	must	bust	**33**	way	say	day	**46**	mark	lark	hark
	sang	bang	fang		just	dust	gust		may	pay	gay		dark	park	bark
**8**	till	will	bill	**21**	pan	pack	pad	**34**	best	west	nest	**47**	heat	heave	heath
	fill	hill	kill		path	pass	pat		test	rest	vest		heal	heap	hear
**9**	man	mass	map	**22**	din	did	dip	**35**	pace	page	pay	**48**	den	then	men
	math	mat	mad		dig	dim	dill		pave	pace	pane		ten	hen	pen
**10**	male	sale	bale	**23**	sit	wit	bit	**36**	back	bad	bat	**49**	thaw	paw	raw
	tale	gale	pale		hit	fit	kit		bash	ban	bath		law	saw	jaw
**11**	safe	sale	save	**24**	sin	tin	pin	**37**	top	pop	cop	**50**	bead	beam	beak
	same	sane	sake		din	fin	win		shop	mop	hop		bean	beat	beach
**12**	peat	peak	peal	**25**	tear	teal	team	**38**	wig	rig	fig				
	peace	peach	peas		teak	tease	teach		dig	pig	big		**SCORE**		
**13**	kid	kin	kit	**26**	dent	went	rent	**39**	tab	tam	tack				
	king	kick	kill		sent	bent	tent		tan	tap	tang				
