# Supplementary material for: HALO CleanSpace PAPR evaluation: Communication, respiratory protection, and usability
Source: Infect Control Hosp Epidemiol. 2022 Apr 1;44(2):295–301. doi: 10.1017/ice.2022.71 (PMC9929704; doi:10.1017/ice.2022.71)
Supplement: Supplementary file 1 [file S0899823X2200071Xsup.zip › S0899823X2200071Xsup002.pdf]

Appendix 2: Commonly Used Medical Phrases Sample

1. Start chest compression
2. Oxygen away
3. Give IM adrenaline
4. I need ultrasound please
5. Pass me the Laderal bag please
6. Does the patient have any allergies
7. Has the patient been paralyzed
8. Can you run a blood gas
9. I have altered the MET criteria
10. Please keep the set up sterile
11. I need a red guedel
12. Press the emergency buzzer
13. Put the BIS on please
14. Sit the patient right up for CXR
15. Tape the eyes please
16. Give a fluid bolus please
17. Blood pressure too high
18. I need the suction please
19. I cannot ventilate the patient
20. 12 lead ECG please
