# Supplementary material for: HALO CleanSpace PAPR evaluation: Communication, respiratory protection, and usability
Source: Infect Control Hosp Epidemiol. 2022 Apr 1;44(2):295–301. doi: 10.1017/ice.2022.71 (PMC9929704; doi:10.1017/ice.2022.71)
Supplement: Supplementary file 1 [file S0899823X2200071Xsup.zip › S0899823X2200071Xsup003.pdf]

Appendix 4: Cleanspace HALO usability and comfort survey

We would like to know what your experience is with the HALO device.

1. How many times have you used the HALO device in the past, excluding this evaluation project (tick one)?
    - Never
    - 1-5
    - 6-10
    - 11-15
    - >15

2. How would you rate the seal of the device? (one option only)
    - poor
    - adequate
    - good
    - very good
    - excellent

3. How would you describe the ease of use when donning? (one option only)
    - easy
    - somewhat difficult
    - difficult

4. How would you describe the ease of use when doffing? (one option only)
    - easy
    - somewhat difficult
    - difficult

5. How would you describe the firmness of fit on the face? (one option only)
    - too tight
    - about right
    - too loose

6. How would you describe the breathability? (one option only)
    - poor
    - average
    - good

7. How would rate the communication performance of the device (one option only)
    - poor
    - average
    - good

8. Have you ever had any of the following problems with the HALO device? (Yes or no for each option)
    - Eye irritation
    - Skin irritation or rash
    - Anxiety/claustrophobia
    - Too heavy
    - Too noisy
    - Significant pressure areas that have limited your duration of use of the mask
        i. Nose
        ii. Cheeks
        iii. Ears
        iv. Chin
        v. Other (please specify)

9. How would rate the overall comfort (Feel) of the HALO device (one option only)
    - poor
    - adequate
    - good
    - very good
    - excellent

10. What is your overall assessment of this type of N95 mask? (one option only)
    - poor
    - adequate
    - good
    - very good
    - excellent

11. Would you prefer to use the HALO device rather than a N95 mask?
    - Yes or No

12. Are you willing to use the HALO device if it is supplied by the hospital?
    - Yes or No

13. Please add any other free text comments about this device:
