# Supplementary material for: HALO CleanSpace PAPR evaluation: Communication, respiratory protection, and usability
Source: Infect Control Hosp Epidemiol. 2022 Apr 1;44(2):295–301. doi: 10.1017/ice.2022.71 (PMC9929704; doi:10.1017/ice.2022.71)
Supplement: Supplementary file 1 [file S0899823X2200071Xsup.zip › S0899823X2200071Xsup004.pdf]

Appendix 1: Modified Rhyme Test single-syllable word list sample

1. The word is **lick**
2. The word is **beat**
3. The word is **puff**
4. The word is **cook**
5. The word is **tip**
6. The word is **rave**
7. The word is **hang**
8. The word is **till**
9. The word is **math**
10. The word is **sale**
11. The word is **same**
12. The word is **peal**
13. The word is **kit**
14. The word is **sat**
15. The word is **sin**
16. The word is **gold**
17. The word is **buff**
18. The word is **lay**
19. The word is **nun**
20. The word is **must**
21. The word is **pad**
22. The word is **din**
23. The word is **sit**
24. The word is **win**
25. The word is **teak**

26. The word is **dent**
27. The word is **sub**
28. The word is **led**
29. The word is **tot**
30. The word is **dub**
31. The word is **pip**
32. The word is **seen**
33. The word is **way**
34. The word is **west**
35. The word is **pace**
36. The word is **bat**
37. The word is **mop**
38. The word is **big**
39. The word is **tab**
40. The word is **case**
41. The word is **name**
42. The word is **soil**
43. The word is **fin**
44. The word is **cuff**
45. The word is **heal**
46. The word is **hark**
47. The word is **heat**
48. The word is **then**
49. The word is **law**
50. The word is **bean**
